# Research publications and global manufacture of veterinary vaccines against avian influenza A (2019-2023)

**DOI:** 10.3389/fvets.2025.1394675

**Published:** 2025-03-12

**Authors:** Aníbal Domínguez-Odio, Ernesto Rodríguez-Martínez, Mayelin Paneque Zayas, Daniel Leonardo Cala-Delgado

**Affiliations:** ^1^Dirección de Ciencia e Innovación. Grupo Empresarial LABIOFAM, Boyeros, Cuba; ^2^Department of Mathematics, Instituto de Cibernética, Matemática y Física, La Habana, Cuba; ^3^Animal Science Research Group, Universidad Cooperativa de Colombia, Sede Bucaramanga, Bucaramanga, Colombia

**Keywords:** Avian influenza virus, vaccine, strain, adjuvant, livestock, pets, technological surveillance

## Abstract

The characteristics of the avian influenza virus and its worldwide spread have led to intense and unprecedented scientific activity and industrial production for preventive veterinary vaccines. However, knowledge gaps remain regarding the best strategies to prevent epidemiological events in the future. In this context, the present study aimed to provide a global analysis on the scientific and industrial production of avian influenza type A vaccines for farm animals and pets during the period 2019 2023. The Scopus database was used as the primary source of information (12,162 keywords, 2,437 scientific articles, 659 academic journals, and 46 countries) for the academic analysis, while technical information posted on official institutional websites (136 commercial formulations, 24 vaccines manufacturers, and 17 countries) was collected to conduct the industrial analysis. 3,045, 25.0%) exhibited the highest levels of co-occurrence in the sciences; the journal Vaccine was the most productive in terms of articles (11.8%, 288/2,437), and the countries with the most publications were the USA (25.5%, 622/2,437) and China (23.1%, 564/2,437). The most internationally marketed vaccines were inactivated (86.0%, 117/136), avian (47.1%, 64/136), and combined (52.2%, 71/136) vaccines as well as those containing Newcastle antigens (38.0%, 27/71). In conclusion, the study demonstrated the fundamental role of classical production methods (based on the use of the whole pathogen) in avian influenza A research and the production of veterinary vaccines.

## 1 Introduction

Avian influenza is currently a major animal health concern worldwide. It is a zoonotic disease that spread among wild/domestic birds and mammals (terrestrial or aquatic), including cattle, and humans (1, 2). The virus is highly contagious and is transmitted between infectious and susceptible animals, principally via the respiratory route, therefore it is considered a mandatory notification sickness by the Word Organization for Animal Health (WOAH).

The severity of the clinical effects usually varies according to the type of virus-those with low pathogenicity result in low mortality rates, while the highly pathogenic ones can cross the respiratory and intestinal barriers, spread through the bloodstream, damage all tissues, and lead to high mortality rates (3–5). In all cases, vaccines are considered as additional preventive tools to limit the spread of the virus, reduce the severity of the disease, reduce the morbidity rate during outbreaks, improve the recovery of the sick animal and reduce the significant damage it causes, including zoonotic damage (6, 7).

Anti-influenza immunopreventive formulations have some limitations, such as the low capacity for generating cross-immunity and effectiveness dependent on the antigenic closeness between circulating and vaccine strains (8). The uncertainty regarding this last aspect is based on the abrupt genetic and antigenic changes of the etiological agent, caused by its ability to replicate without mutation protection mechanisms and by the recombination of genomes between strains (9–11). The hemagglutinin and neuraminidase contributes to this problem (12, 13), as subtle changes in the amino acids in its structure can lead to the emergence of new lineages, with renewed capacity to cross the barrier between species (10, 14, 15), and escape the defense of the host. These frequent mutations of the virus are the main limiting factor for the development of an effective vaccination (16).

Many companies and governments are considering research and development of veterinary vaccines against avian influenza type A despite these difficulties (17). Although this official projection can achieve technological sovereignty and self-sufficiency of vaccination programs, it also requires strategies to produce safe, effective, and stable high-yielding vaccines with high yields (18). Therefore, the WOAH has made recommendations to interested nations (19), providing access to production technology adaptable to their epidemiological realities (20). However, all the information mentioned is not sufficient to achieve success, as it is necessary to know the scientific-commercial environment surrounding these vaccines, the emerging production technologies, and the new diversity of adjuvants (21).

Unfortunately, this knowledge is often unavailable to researchers and entrepreneurs as it is dispersed among various scientific fields and is not very attainable. In view of these difficulties, technological surveillance studies are a useful tool for obtaining, concentrating and analyzing existing scientific-technological knowledge, understanding the global commercial environment and facilitating strategic decision-making in the industrial sector. Based on this, the present study aimed to identify research, production technology and commercial trends of veterinary vaccines against avian influenza A in farm animals and pets during the period 2019–2023 globally.

## 2 Materials and methods

This study was descriptive in nature, employing a methodology that combines qualitative and quantitative methods. It involved retrospective bibliometric research covering the last 5 years (2019–2023), along with additional cross-sectional market studies conducted in 2023. The study was conducted from September to November 2023, and in no case was the use of experimental animals required.

### 2.1 Search for key terms and data extraction

#### 2.1.1 Bibliometric study

Our analysis was restricted to scientific articles written in English, published in the last 5 years (January 1, 2019–October 31, 2023) and indexed in the Scopus database, which constitutes the largest database of peer-reviewed scientific literature worldwide (22).The retrospective analysis of the most recent literature on avian influenza immunoprevention in avian, equine, swine, and canine species exclusively took into account original article, systematic reviews, data analyses and short research reports.

The identification and selection of relevant publications was performed independently by two researchers (AD-O and ER-M) to enhance the methodological strength, and disagreements were resolved in public discussions with a third member of the research team (DLC-D). The fields ‘title’, ‘abstract’ and ‘keywords’ as well as the main terms and their grammatical variants used in the English scientific literature to refer to avian influenza (avian flu, bird flu, equine flu, swine flu, and canine flu) and selected animal species (bird, poultry, chicken, turkey, duck, horse, pig, porcine, hog, dog, and pets). Careful checks were performed to ensure that the initial search was as sensitive as possible and that any restrictions increased specificity without compromising sensitivity.

The processes for searching, selecting, and collecting the articles in the databases were conducted using keywords and Boolean connectors. One example search is: TITLE-ABS-KEY (“avian influenza virus” OR “avian influenza” OR “equine influenza” OR “swine influenza” OR “canine influenza”) AND TITLE-ABS-KEY (“influenza vaccine” OR “vaccine” OR “vaccines” OR “combined vaccine”). Several attributes were extracted from the selected articles, including frequently used keywords, principal author’s, country or region of origin, and journal that published the article. Articles with corrections, book and chapter reviews, news, discussions and retracted publications were excluded.

### 2.2 Market study

#### 2.2.1 Data extraction

To identify candidate veterinary vaccine manufacturers, the database prepared by the scientific-technological observatory belonging to the LABIOFAM Business Group was initially consulted. The final list was defined through a two-stage selection process. In the first step, two reviewers (ADO and MP) independently identified candidates (*n* = 45) involved in the research, development, manufacture, export and marketing of veterinary vaccines. A conservative approach was adopted in this step and all vaccine manufacturers selected by at least one of the reviewers were retained for the next step. Geographic location and type of ownership (public or private) were not exclusion criteria.

The second step was based on the commercial availability of avian influenza A vaccines for use in poultry, equines, swine or canines. Based on this criterion, 13 companies that stated on their official websites that they declared not to have a formulation available were excluded from the study. The technical contents provided by the manufacturers that could characterize the vaccines were also analyzed, for example: production technology, adjuvants and antigen used in each formulation. The absence of an explicit description in Spanish or English excluded eight vaccines manufacturers. Finally, both reviewers discussed their respective final selections until a consensus was reached on each company (*n* = 24). In the absence of consensus, the opinion of a third reviewer (DLC-D) was solicited.

### 2.3 Data analysis and visualization

The complete records of the retrieved articles were downloaded or manually entered into a Microsoft Excel spreadsheet (Microsoft Corporation, Redmond, WA, USA), and any discrepancy or disagreement was discussed and resolved with other authors. For the metric analysis of the extensive scientific production obtained (keywords: 12,162, academic journals: 659, countries: 46), a minimum of seven coincidences was established in order to obtain legible co-occurrence maps and visualize the most relevant thematic nodes. The retrieved records were exported to the EndNote X9 bibliographic reference manager, where duplicates were eliminated.

To explore the co-occurrence relationships of keywords, academic journals and countries together with their respective scientific collaboration networks, the software tool VOSviewer 1.6.18, developed by Jan van Eck and Ludo Waltman of Leiden Nees University, was used. Knowledge maps based on academic articles were represented by circular nodes (*n* = 68), connecting links and colors for the different clustering clusters. The intensity of relationships between nodes was estimated as a function of their proximity and the thickness of the connecting lines: close labels and thick connecting lines were interpreted as high co-occurrence rate (23).

The public platform SCImago Journal & Country Rank,^1^ developed by the SCImago group based on information from the Scopus database, was used to assess the quality of scientific articles (*n* = 2,437) on veterinary vaccines against avian influenza published in academic journals from 2019 to 2023. The quartile in which each journal is located (Q1-Q4), the SCImago Journal Rank index (indicator of the prestige of academic journals that takes into account the number of citations received and the prestige of the journals from which the citations originate), and h-index were considered (number of h citations a journal has received in other publications).

The market analysis included technical information on 136 commercial formulations from 24 company in 17 countries. The data were previously registered and coded in an electronic Microsoft Excel spreadsheet (2019) for organizational and control purposes. The variables-animal species, production technology (traditional or modern), type of vaccines (inactivated, live attenuated, recombinant, subunit and RNA vaccines), adjuvants, vaccine strains (subtypes and host of origin), and types of formulation (monovalent, polyvalent, and combined)-were summarized and expressed in absolute (*n*) and relative frequency (%). Groups of formulations with similar profiles were subsequently identified, and the existing associations between their attributes were determined through multiple correspondence analyses using the FactoMineR software package, version 4.3.1 (USA).

The selected variables for exploring possible relationships were production technology, formulation, and animal species. Subsequently, significant differences in the frequencies of vaccine strain use (high and low pathogenicity) and types of formulation across animal species (avian, swine, equine, and canine) were identified using the independent chi-square test. Once it was confirmed that the frequencies of use were not homogeneous (*p*-value <0.05), the pairwise chi-square test was employed using the R package, version 0.7.2 (USA). The *p*-values obtained were adjusted using the Holm–Bonferroni method to reduce errors incurred when performing multiple tests.

## 3 Results

### 3.1 Scientific trend

#### 3.1.1 Knowledge map

**Figure 1:**
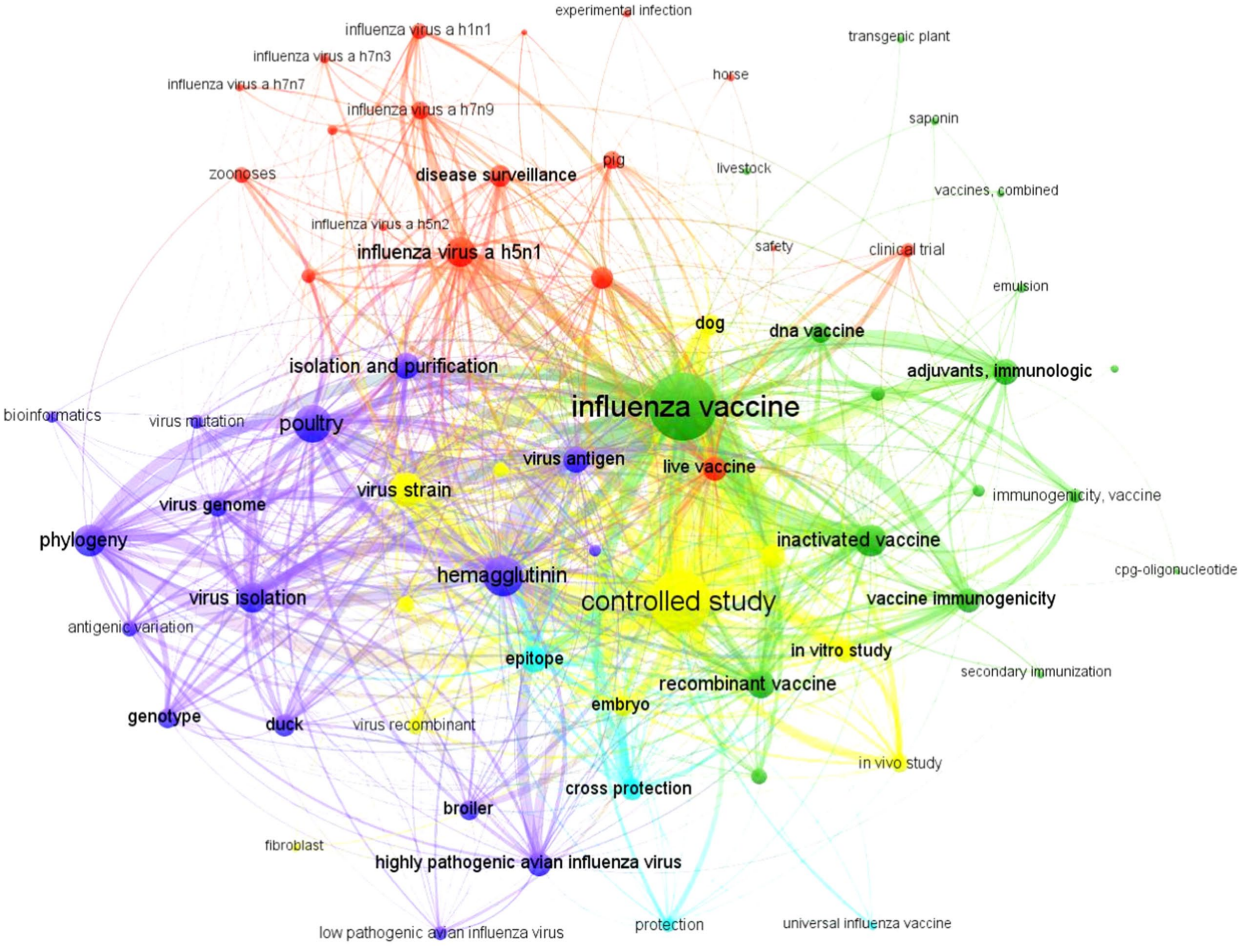
Co-occurrence of keywords and links established between the thematic areas dedicated to veterinary vaccines against avian influenza type A during the period 2019–2023. Source: Authors’ own elaboration with VOSviewer^®^ v1.6.18 software based on data obtained from Scopus^®^.

The complex co-occurrence network or knowledge map, consisting of keywords (*n* = 12,162) found in scientific articles (*n* = 2,437) on the veterinary prevention of avian influenza, is shown in Figure 1. The nodes visualized (*n* = 68) using the VOSViewer clustering algorithm formed five important clusters (green, yellow, dark blue, red, and light blue), all with intense collaboration links between them. The most frequently used descriptors were “influenza vaccine” and exhibited the highest frequency of use in specialized publications (50.5%, *n* = 6,144) during the period 2019–2023 and was located near the center of the map.

The thick and numerous connecting lines displayed indicate high levels of co-occurrences with numerous nodes, highlighting the controlled study (25.0%, *n* = 3,045), inactivated vaccine (11.8%, *n* = 1,440), immunological adjuvants (9.0%, *n* = 1,100), and the recombinant vaccine (*n* = 1,005, 8.2%). It is important to note the closeness and strong co-occurrence links it established with other nodes outside its thematic cluster, such as live vaccine (red, *n* = 720) and canine vaccine (yellow, *n* = 510), while with horse (red, *n* = 19) it maintained little links and co-occurrence.

Cluster 1 (green, *n* = 21, 30.9%) exhibited the greatest strength and breadth in the visualized knowledge map. It focused on inactivated, recombinant, and DNA vaccines; adjuvants; and study of immunogenicity. Meanwhile, cluster 2 (yellow, *n* = 19, 27.9%) was associated with controlled studies, field strains, specific-pathogen-free chicken embryos, *in vitro* and *in vivo* studies, and dogs. Cluster 3 (dark blue, *n* = 14, 20.6%) was particularly related to hemagglutinin, virus antigen, virus isolation, virus genome, phylogeny study, genotype, and avian species. Cluster 4 (red, *n* = 10, 14.7%) was linked to disease surveillance, influenza A subtype H5N1, H5N2, H7N7, H7N3, H7N9, H1N1, zoonoses, pig, clinical trials, safety, and live vaccines. Cluster 5 (light blue, *n* = 4, 5.9%) had the least representation among all and was located on the periphery of the map, focusing on research on cross-protection, epitopes, and universal vaccines (Figure 1).

#### 3.1.2 Publication sources

**Figure 2:**
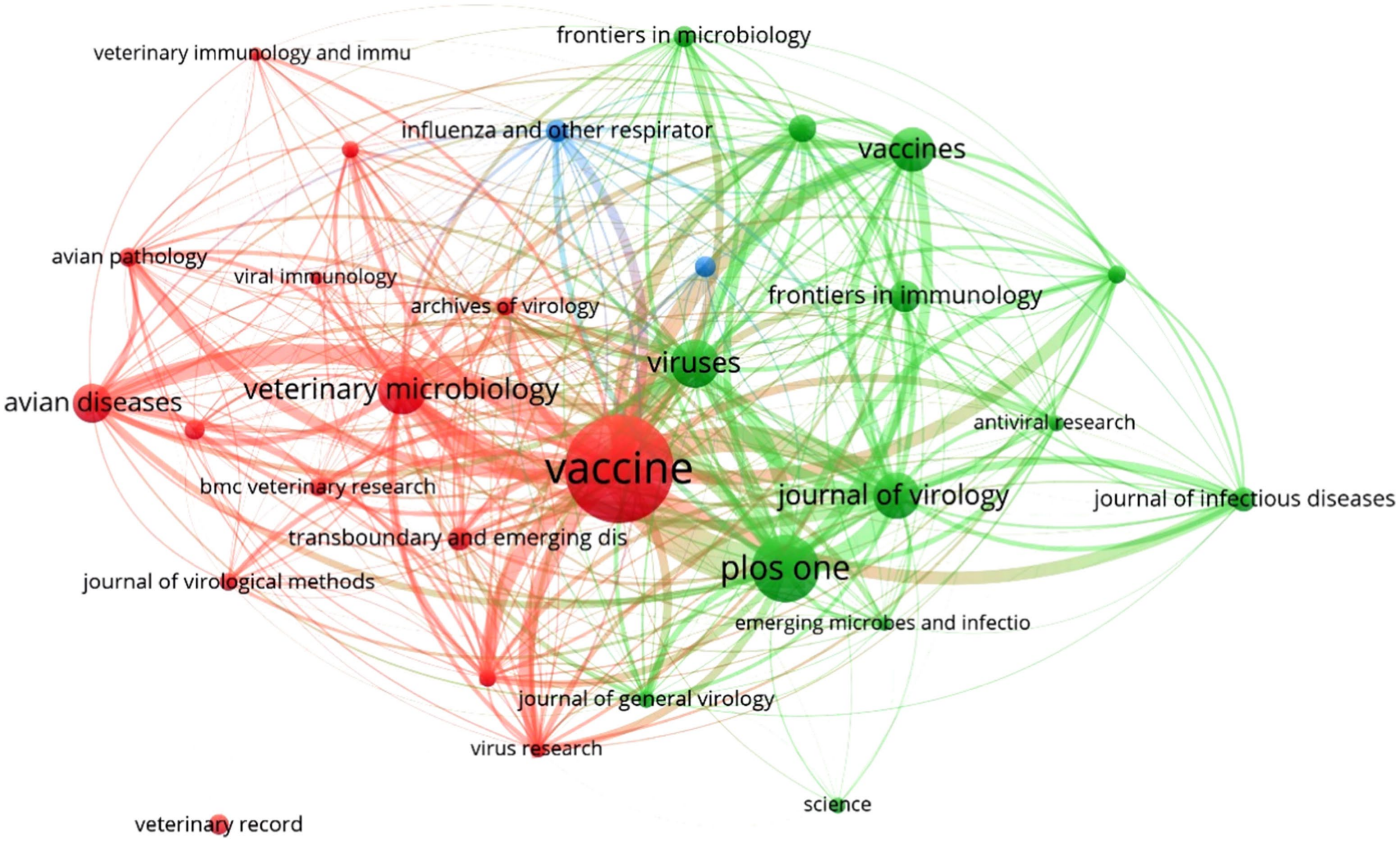
Most productive scientific journals on veterinary vaccines against avian influenza type A during the period 2019–2023. Source: Authors’ own elaboration with VOSviewer^®^ v1.6.18 software based on data obtained from Scopus^®^.

The associative analysis between relevant academic journals on the topic of vaccines against avian influenza (*n* = 659), their publications (*n* = 2,437), and the respective citations generated (*n* = 23,332) showed that only 3.6% (*n* = 24) had the highest productivity (Figure 2). When arranged in descending order based on their impact, the latter showed a high scientific level as a whole, where 50.0% (12/24) were Q1, 33.3% (8/24) were Q2, 16.7% (4/24) were Q3, and none of them were classified as Q4. From a country standpoint, 37.5% (9/24) of the most productive journals were from the United Kingdom, followed by the Netherlands (25.0%, 6/24), the United States (20.8%, 5/24), Switzerland (12.5%, 3/24), and Austria (4.2%, 1/24), thus demonstrating the dominance of the Anglo-Saxon language in the production and impact of scientific research on veterinary avian influenza vaccines.

The most active journal (*n* = 288, 11.8%) and with the highest citation levels (*n* = 2,899, 12.4%) in this topic during the period 2019–2022 was Vaccine, which occupied the central core of the bibliometric map. It was followed by others of similar prestige and scientific influence, such as PLOS One (153 publications and 1,819 citations), Viruses (88 publications and 529 citations), Veterinary Microbiology (70 publications and 970 citations), and Journal of Virology (63 publications and 1,868 citations). All citations accounted for 27.2% (*n* = 662) of publications worldwide, and 34.7% (*n* = 8,085) of them were linked to the topic of veterinary vaccines against influenza.

The bibliometric analysis also revealed that the five journals had in common their origin in developed countries, their indexations in Q1 and Q2 since 2019, SJR ≥1.49, and h-indices ≥114, in addition to their publication of articles in multiple thematic categories. It is remarkable that among the relevant journals on the subject (*n* = 659), Pakistan Veterinary Journal (Q1, SJR: 0.53, and h-index: 37) and the Veterinary World of India (Q2, SJR: 0.48, and h-index: 48), both from developing countries, had a total of 76 publications and 22 citations during the period 2019–2023.

#### 3.1.3 Academic collaboration

**Figure 3:**
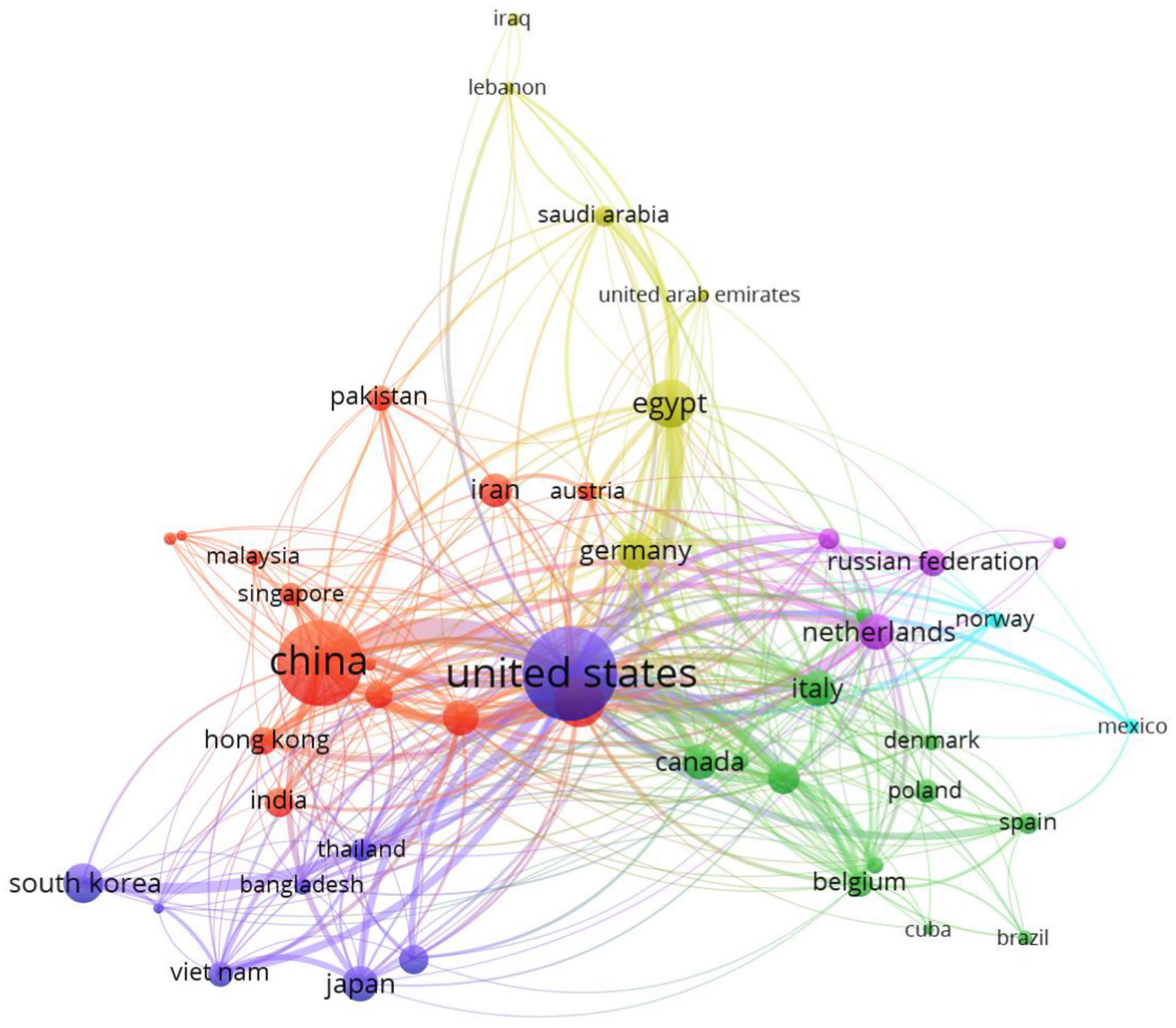
Most productive countries and their collaborative links in terms of veterinary vaccines against avian influenza type A during the period 2019–2023. Source: Authors’ own elaboration with VOSviewer^®^ v1.6.18 software based on data obtained from Scopus^®^.

Figure 3 shows the countries that participated in publications on veterinary vaccines against avian influenza (*n* = 46) and their collaborative links (*n* = 391). Distribution based on country revealed six clusters: the United States (dark blue) in the center, surrounded by China (red), Egypt (yellow), the Netherlands (purple), Italy (green), and Mexico (light blue). The six countries mentioned above accounted for 66.3% (*n* = 1,616) of all scientific publications related to the topic (*n* = 2,437) during the period 2019–2023, with the United States as the main contributor (*n* = 622, 25.5%), followed by China (23.1%, *n* = 564) and Egypt (6.6%, *n* = 160).

A similar hierarchical order was observed when analyzing the international collaboration groups, led once again by the United States (*n* = 36 countries) and China (*n* = 28 countries). However, the scientific network had significant participation from six Asian countries (South Korea, Vietnam, Japan, India, Pakistan and Iran), seven European countries (Belgium, Poland, Spain, Italy, Germany, the Netherlands and Russia), Canada and Egypt.

### 3.2 Business trend

#### 3.2.1 Characterization of the selected companies

Technical information from 136 vaccines developed, produced, and marketed by 24 biopharmaceutical companies was used to characterize the global market for veterinary vaccines against avian influenza type A in avian, equine, swine, and canine species during 2023. Based on the geolocation declared for their headquarters, America was the best represented, with 13 companies (Avimex, Laboratorios Avilab, BioChemiq, Elanco, Farvet, Instituto Rosenbusch S.A., Lapisa, Microsules, MSD Animal Health/Merck & Co., Sanfer, Veterquimica, Viren S.A., and Zoetis), followed, in descending order, by Europe with six (Bioveta Ltd., Boehringer Ingelheim, Ceva Santé Animale, Dechra Pharmaceutical, Laboratorios Hipra S.A., and Vaxxinova), Asia with four (Navetco, Qilu Animal Health, Razi Vaccine & Serum Research Institute, and Stavropol Biofabrika), and Africa with one (Mevac). Distribution based on country showed that Mexico was leading with five companies, followed by the United States of America with three, Argentina with two, and the remaining participating countries such as the Czech Republic, Germany, France, England, Peru, Spain, Egypt, Uruguay, Vietnam, China, Iran, Russia, the Netherlands, and Chile, each with one.

**Figure 4:**
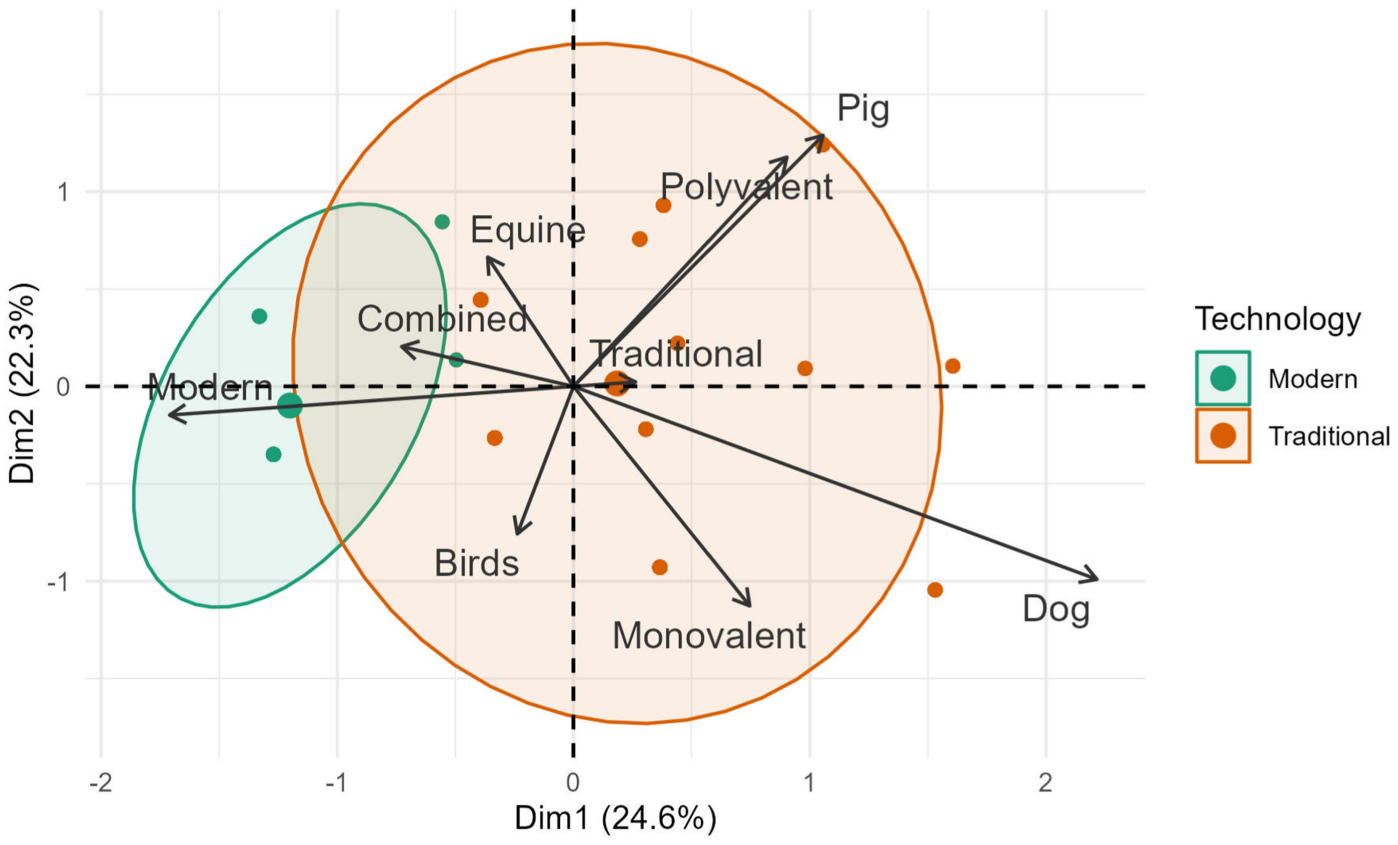
Global manufacture of veterinary avian influenza vaccines grouped by production technology, animal species and formulation. Source: Scientific-technological observatory of the Grupo Empresarial LABIOFAM.

It was found that 100% of the 24 companies selected showed a strong association in the use of similar technologies to produce veterinary vaccines against avian influenza (Figure 4). Vaccines manufactured with traditional technology (inactivated) were the most common on the international veterinary market (86.0%, 117/136), followed in decreasing order by modern vaccines (recombinant, subunit and RNA vaccines) with 13.9% (19/136).

In general terms, 98.5% (134/136) of commercial veterinary avian influenza vaccines were found to be highly dependent on adjuvants. The strategy for industrial use of adjuvants was diverse: 81.3% (109/134) of the formulations contained a single immunostimulant, while the remaining 18.7% (25/134) included mixtures of two or more. The catalog of licensed adjuvants was dominated by oily compounds (52.2%, 70/134), commercial mixtures such as Havlogen®, Amphigen®, MetaStim® and Carbimmune® (18.7%, 25/134), compounds of natural origin such as saponins, polymers and carbomers (16.4%, 22/134) and, finally, aluminum hydroxide (12.7%, 17/134).

#### 3.2.2 Animal species

A second distinctive feature of the avian influenza veterinary vaccine market was the existence of significant differences between observed and expected frequencies of use across all species analyzed, suggesting that influenza vaccine use varies significantly by animal species (Table 1). In particular, there were two extremes of higher and lower frequency of use; birds showed significant values of higher association, while canines use it the least.

**Table 1:** Global production of avian influenza veterinary vaccines grouped by farm animal and pets.

Species	Observed values	Estimated	Statistic	Degrees of freedom	*p*-value	Adjusted *p*-value*
Avian	64	33.75	36.1506	1	< 0.0001	< 0.0001
Swine	18	33.75	9.8000	1	0.0018	0.0035
Equine	47	33.75	6.9358	1	0.0084	0.0084
Canine	7	33.75	0.4228	1	< 0.0001	< 0.0001
General total	136					

^*^values adjusted using the Holm–Bonferroni method.

**Figure 5:**
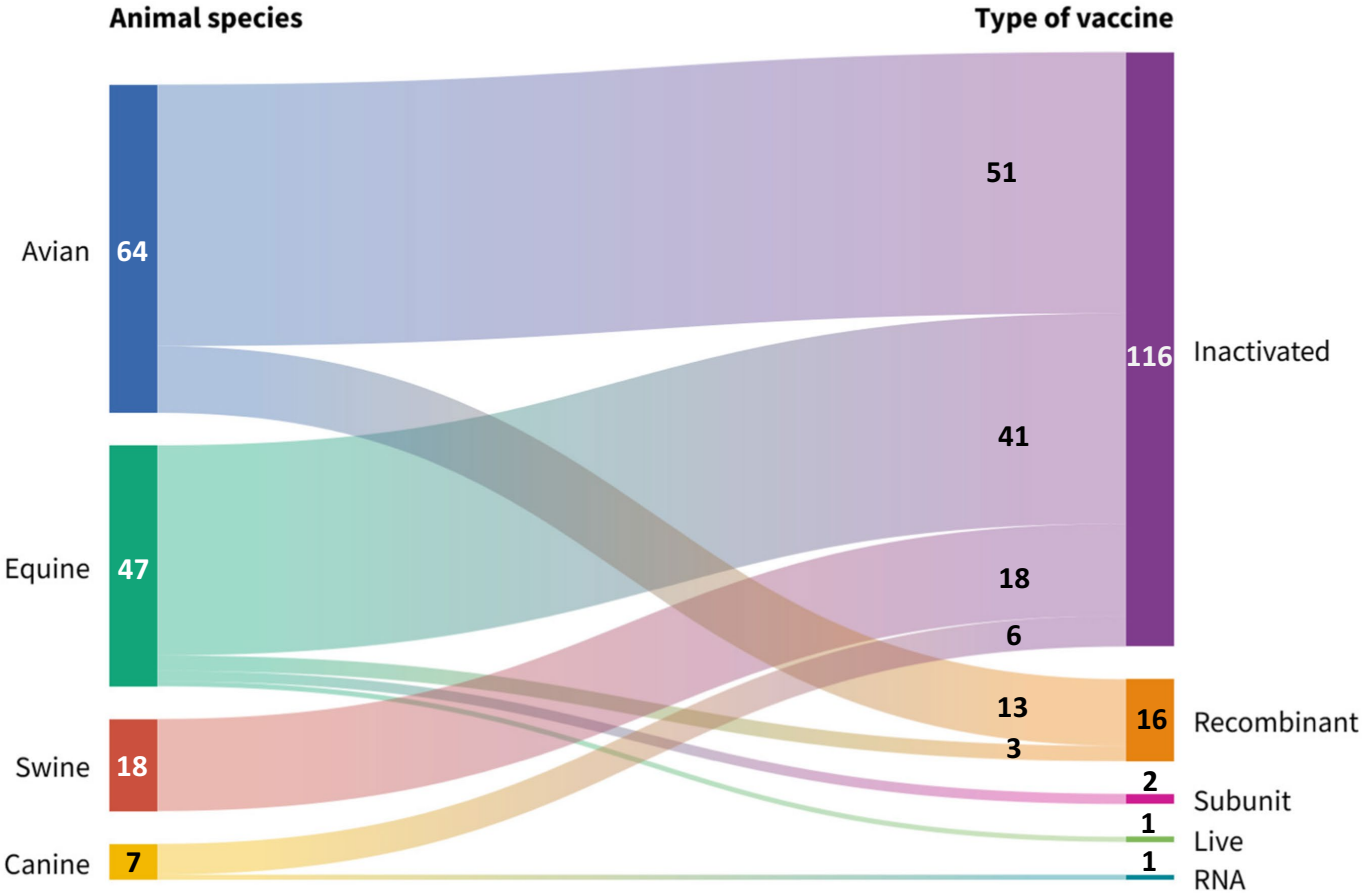
Globally reported production technologies for manufacturing veterinary avian influenza vaccines, grouped by animal species. Source: Scientific-technological observatory of the Grupo Empresarial LABIOFAM.

All species included in the study have inactivated vaccines for immunoprotection (85.2%, 116/136), but only the canine has a commercial vaccine based on RNA technology (Nobivac® NXT Canine Flu, H3N2). The avian species was the most favored by the companies included in the study (47.1%, 64/136), followed, in descending order, by the equine (34.6%, 47/136), porcine (13.2%, 18/136) and canine species. (5.1%, 7/136). Figure 5 shows that equids are the animal species with the greater diversity of vaccines, of which 87.2% are inactivated (41/47), 6.4% (3/47) are recombinant, 4.3% (2/47) are subunit vaccines, and only 2.1% (1/47) are live attenuated vaccines. (Flu Avert®, H3N8).

#### 3.2.3 Commercial vaccine strains

Regarding influenza strains used for industrial purposes, Table 2 summarizes the observed and expected frequencies according to animal species during 2023. The absence of homogeneity at the international level was observed. All species showed significant differences in the use of low and high pathogenicity strains, except for canines. Avian vaccines used approximately 3.0 times more highly pathogenic strains in their formulation, while swine vaccines used 24.0 times more low pathogenic strains. This indicates that the selection of strain type may be influenced by the animal species and its context of use.

**Table 2:** Low and high pathogenicity strains available for global manufacture of avian influenza vaccines distributed by farm animal and pets.

Species	Observed values		Degrees of freedom	*p*-value	Adjusted *p*-value^*^
	Low pathogenicity	High pathogenicity			
Avian	21	64	1	< 0.0001	< 0.0001
Swine	48	2	1	< 0.0001	< 0.0001
Equine	46	10	1	0.0004	0.0008
Canine	9	0	1	0.0582	0.0582

^*^values adjusted using the Holm–Bonferroni method.

**Figure 6:**
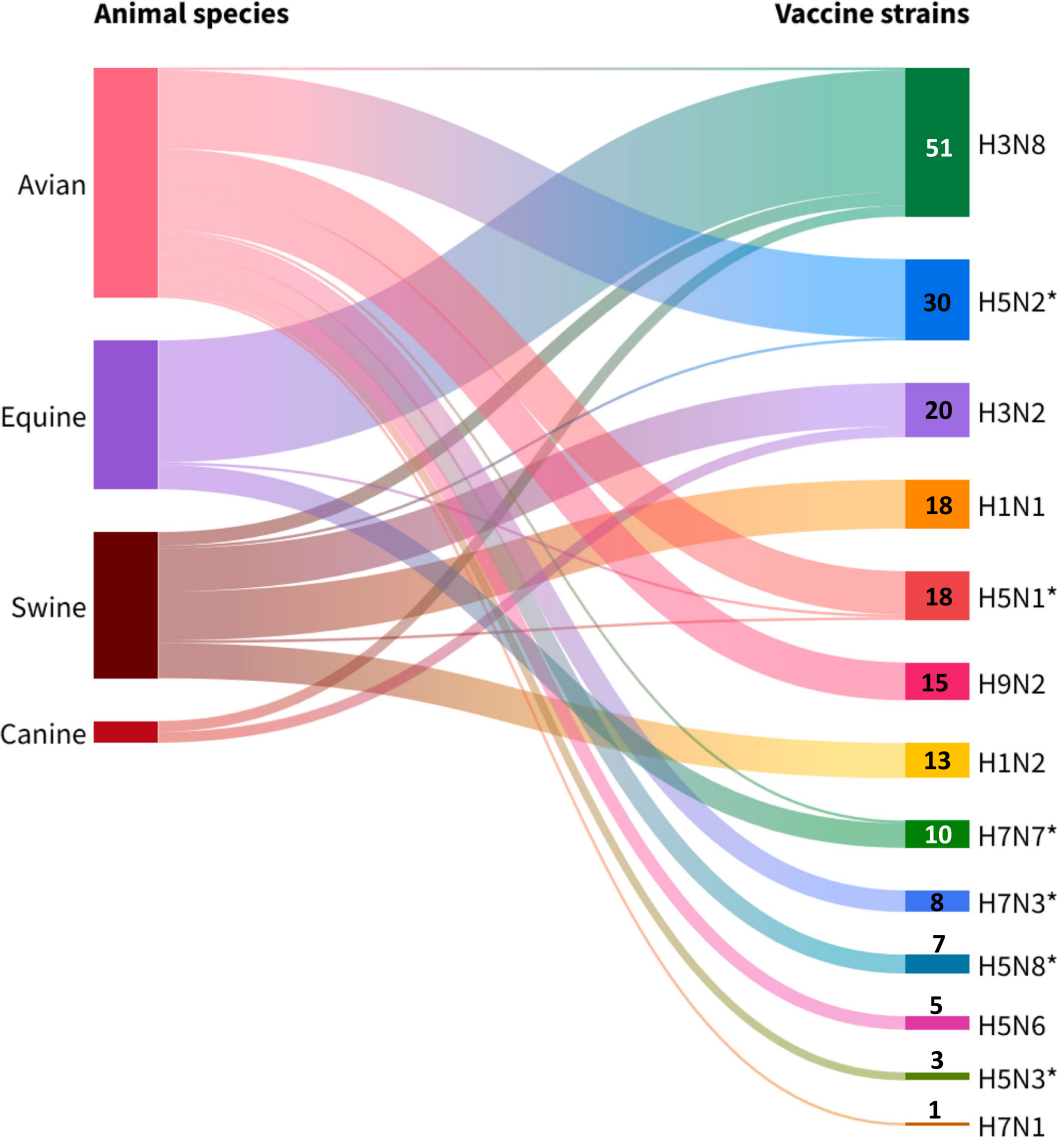
Avian influenza strains isolated from different animal species available for global veterinary vaccine production. Note: *highly pathogenic vaccine strains. Source: Scientific-technological observatory of the Grupo Empresarial LABIOFAM.

A diverse range of subtypes (*n* = 13) used in the production of commercial vaccines was identified (Figure 6). The H5Nx subtype (H5N1, H5N2, H5N3, H5N6 and H5N8) showed the highest antigenic diversity (38.5%, 5/13), followed by the H7Nx subtype (H7N1, H7N3 and H7N7) with 23.1% (3/13). Avian vaccines were found to have the greatest variety of subtypes in their formulation (10/13), followed by porcine (6/13), equine (4/13), and finally canine (2/13).

It was observed that H3N8 (37.5%, 51/136), H5N2 (22.0%, 30/136) and H3N2 (15.4%, 21/136) subtypes were the most commonly used in commercial monovalent, multivalent and combination vaccines. Industrial use of the H3N8 subtype was concentrated in equine vaccines (88.2%, 45/51), while the H5N2 and H3N2 subtypes were mainly reported in poultry (96.6%, 29/30) and swine (76.1%, 16/21) formulations, respectively. Thus, the exclusivity of the H1N1, and H1N2 subtypes for swine vaccines was observed, whereas the H9N2, H7N3, H5N8, H5N6, H5N3, and H7N1 subtypes were reserved only for avian use.

**Figure 7:**
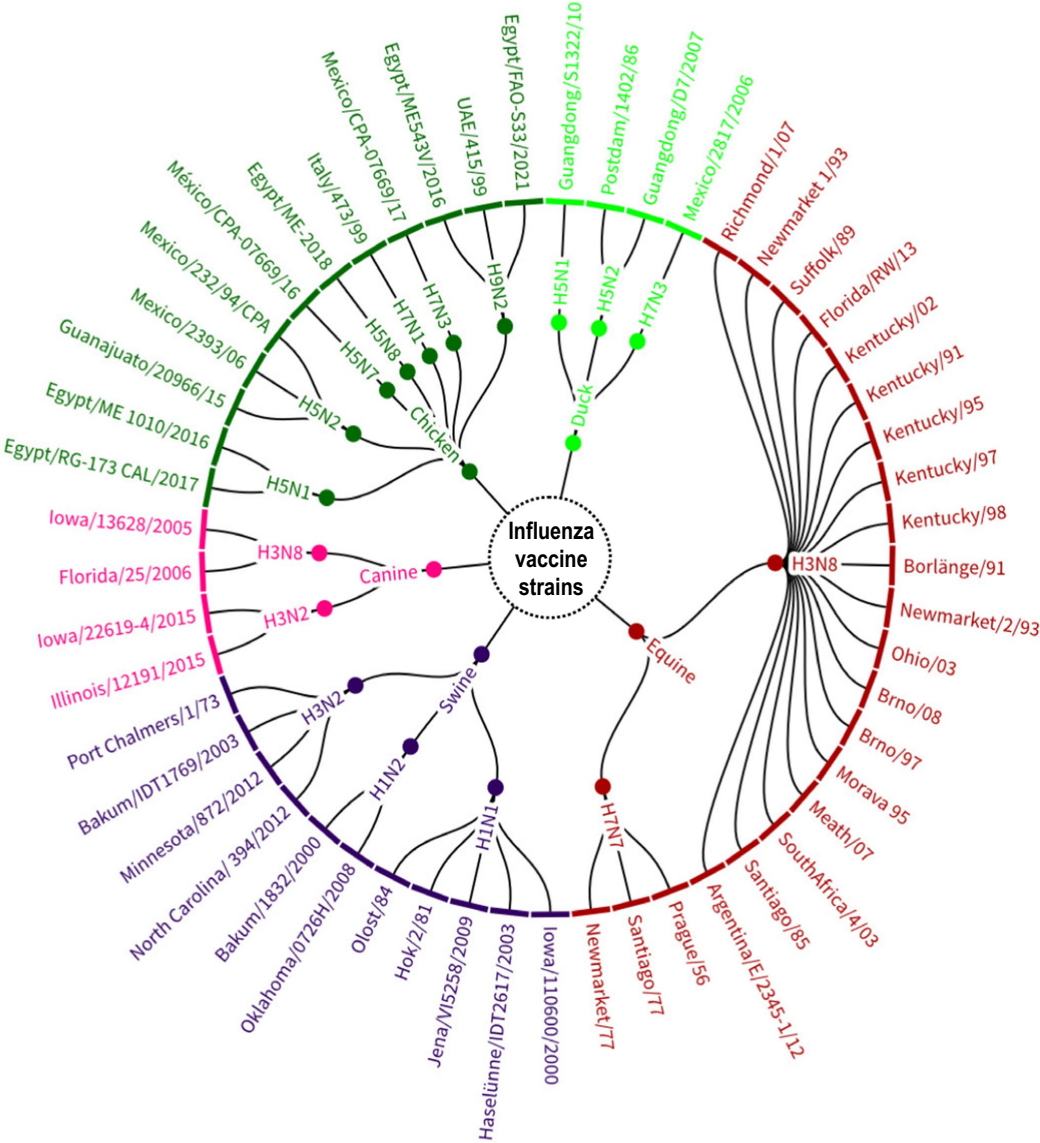
Avian influenza A strains identified in the global manufacture of veterinary vaccines, grouped by subtype and host of origin. Source: Scientific-technological observatory of the Grupo Empresarial LABIOFAM.

The organization of vaccine strains present in commercial vaccines by subtype and animal species also revealed a large genetic diversity (*n* = 53). Figure 7 shows that H3N8 strains isolated from equids were the most diverse (19/53, 35.8%), representing the American and Eurasian lineages as well as the South American, Kentucky and Florida sublineages. In addition, it was the only subtype used for the manufacture of live vaccines (A/Equine 2/Kentucky/91).

#### 3.2.4 Commercial formulations

It was found that 100% (*n* = 24) of the companies contained in their product catalogs vaccines formulated with one (monovalent) or more influenza subtypes (polyvalent), while 66.7% (16/24) produced combined vaccines (mixtures of viral and bacterial antigens). It is important to note that combined vaccines despite being produced by fewer companies dominated the market with 52.2% (71/136), followed by monovalent (29.4%, 40/136) and finally polyvalent (18.4%, 25/136).

Significant differences according to the type of formulation and animal species were identified by the chi-square test (Table 3). Avian monovalent vaccines showed significantly higher frequencies of use compared to the other species. However, equine, swine and avian multivalent vaccines did not differ significantly from canine vaccines, which are the least common in the international market. As for combined vaccines, those for avian and equine species were significantly more widely used than those for canines. This suggests that the choice of influenza vaccine type is significantly influenced by animal species, which could reflect differences in biological needs, management practices or vaccination strategies.

**Table 3:** Manovalent, polyvalent and combined vaccines against avian influenza grouped by farm animal and pets.

Type of formulation	Species	Observed values	Estimated	Statistic	Degrees of freedom	*p*-value	Adjusted *p*-value^*^
Monovalent	Avian	26	11.25	21.0970	1	< 0.0001	< 0.0001
Monovalent	Swine	3	11.25	6.6000	1	0.0102	0.0714
Monovalent	Equine	6	11.25	2.6727	1	0.1020	0.5100
Monovalent	Canine	5	11.25	5.0979	1	0.0248	0.1447
Total		40					
Polyvalent^***^	Avian	6	11.25	2.6727	1	0.1020	0.5100
Polyvalent^***^	Swine	8	11.25	1.0242	1	0.3120	0.6240
Polyvalent^***^	Equine	9	11.25	0.4909	1	0.4840	0.6240
Polyvalent^***^	Canine	2	11.25	8.2970	1	0.0040	0.0318
Total		25					
Combined^**^	Avian	32	11.25	41.7515	1	< 0.0001	< 0.0001
Combined^**^	Swine	7	11.25	1.7515	1	0.1860	0.5580
Combined^**^	Equine	32	11.25	41.7515	1	< 0.0001	< 0.0001
Combined^**^	Canine	0	11.25	12.2727	1	0.0005	0.0041
Total		71					
General total		136					

^*^values adjusted using the Holm–Bonferroni method, ^**^ vaccines formulated using different subtypes of avian influenza type A, ^***^vaccines that combine influenza type A virus antigens with others of viral or bacterial origin.

**Figure 8:**
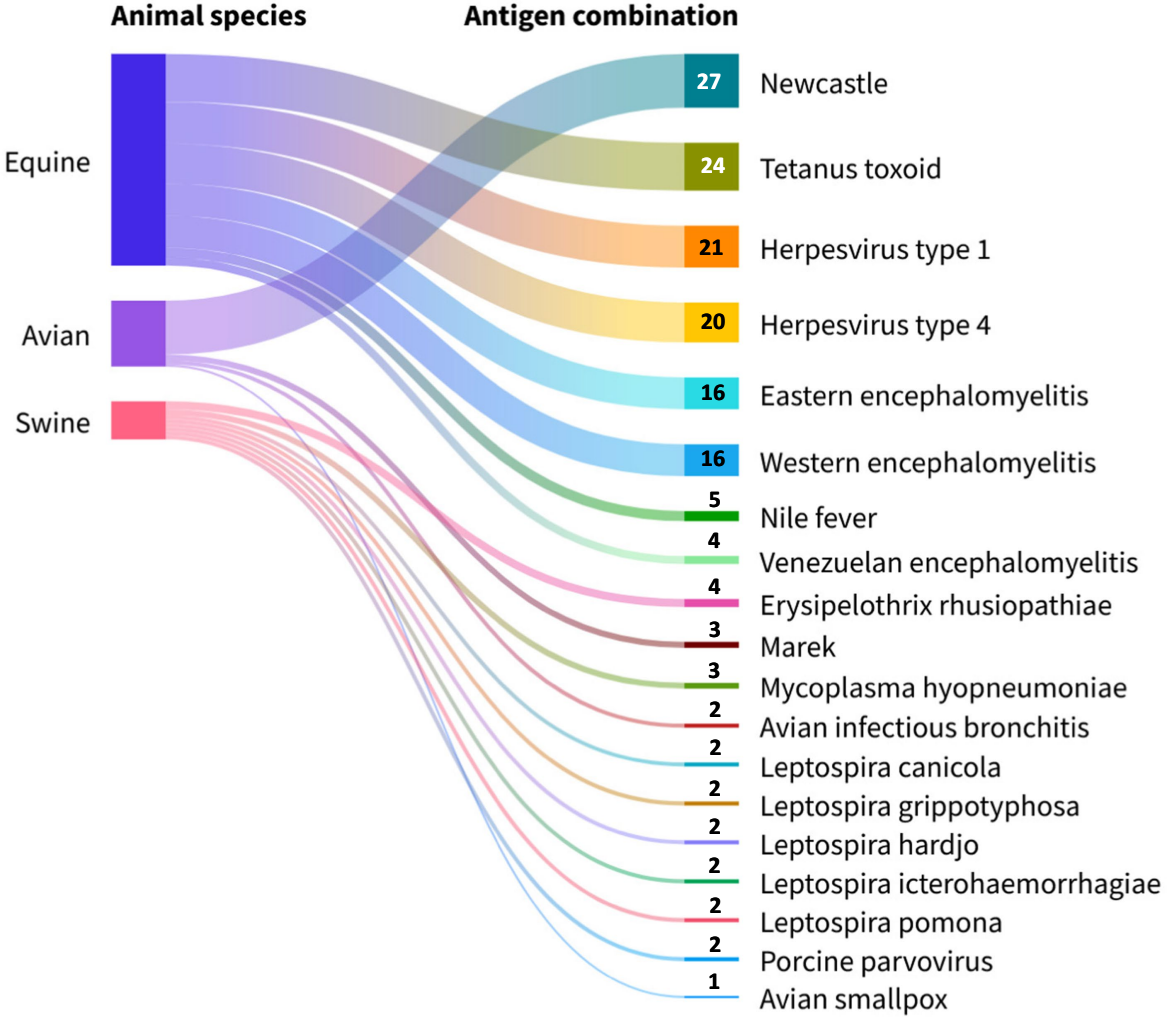
Viral and bacterial antigens included in commercial avian influenza vaccines grouped by animal species. Source: Scientific-technological observatory of the Grupo Empresarial LABIOFAM.

From the point of view of antigenic composition, the formulations combining influenza + Newcastle were the most dominant (38.0%, 27/71), followed by influenza + herpesvirus type 1 (29.5%, 21/71) and influenza + herpesvirus type 4 (28.1%, 20/71). Furthermore, formulations containing bacterial antigens mostly contained tetanus toxoid (33.8%, 24/71), indicated exclusively for equines (Figure 8).

## 4 Discussion

The present academic-commercial analysis of veterinary vaccines against avian influenza confirms that the disease is a priority for the veterinary sector (24). It shows the key role of classical vaccines in reduce the virus shedding in the animal population (25), despite doubts about their efficacy (short-lived immunity), the inability to induce sterilizing immunity and the complications of their use in epidemiological surveillance (7, 8, 26). In this endeavor, science and the veterinary biopharmaceutical industry converge on the same path: inactivated virus vaccine. This common position may be associated to the less clinical signs, less degree of pathological lesions and lower titer of viral excretion observed in birds vaccinated with this type of vaccine (25, 27, 28) and to its rapid development-production.

The popularity achieved by this type of production technology is also supported by decades of application with excellent results and the possibility of each country possibility to manufacture its own formulations from national isolates (24). In this context, classical autogenous vaccines have the advantages of adapting to local health needs, accelerating the response to new influenza subtypes in the field, avoiding vaccinating with outdated strains, achieving national self-sufficiency and low sales prices per dose, and avoiding excessive costs related to long transport times (29, 30). For these reasons, it is expected that science will continue to update local vaccine strains (31) to manufacture and test new inactivated vaccines for chickens/ducks (32), pigs (33) and companion animals (34), depending on the epidemiological conditions in different countries.

The evidence gathered in this research also shows that recombinant vaccines against influenza are the second-best solution studied and marketed globally during the period 2019–2023 to immunoprotect productive species. Such a stance coincides with scientific advances on molecular mechanisms of genomic replication (35), characterization of conserved and variable sites of the viral RNA (36), and, in particular, the expression of hemagglutinin and/or neuraminidase genes in avian smallpox, turkey herpesvirus, Newcastle virus, *Escherichia coli*, and plants (*Nicotiana benthamiana*) (37–40). This latter alternative approach offers significant advantages associated with the ability to grow the plants on a large scale, rapid and efficient production in required quantities, low costs (40) and thermostability of the antigen (39). However, like other vaccines, it does not overcome the challenge of cross-protection and the high risk of occupational exposure from handling the avian influenza antigen remains. The introduction of these plant species for vaccine production requires improved technologies both to minimize the alkaloid (toxic substance) content and to increase the antigen yield. It also requires confined cultivation systems to avoid the possibility of agrochemical contamination, adverse weather conditions, irregular soil composition and pathogen attack (40).

The low presence of modern vaccines (*n* = 16) in the product catalogs issued by the companies studied (*n* = 136), allows inferring that much of the new scientific knowledge remains in the exploratory field, and few are converted into marketable products. Consequently, the role of these formulations in global veterinary health management during the 2019–2023 period remains limited. Changing this reality in the future involves obtaining vaccines capable of effectively stopping virus replication and excretion and generating specific antibodies that do not interfere with the diagnosis of naturally infected animals. Although some progress has been made in this regard (41–43), much work remains to be done; it is necessary to surpass the indicators of safety, efficacy and duration of immunity achieved by traditional vaccines in the field, demonstrate the stability of the synthetic antigen and explore the use of the mucosa (buccal, nasal and ocular) as routes of administration for different animal species.

The need to optimize the efficacy of inactivated vaccines and, the development of new purified, synthetic or recombinant antigens that are specific and well characterized but not very immunogenic, explains, at least in part, the great scientific interest in developing new and potent adjuvants that help to increase the immune response, in addition to generating a rapid decrease in clinical signs (44). The academic projection shown in Figure 1 involves aluminum nanoparticles composed of quaternized chitosan (45), different fractions of purified saponins (46), and natural oils of plant origin (47), among other novel compounds. However, it is expected that the introduction of academic advances into industrial practice will require time and significant financial resources. Its delayed implementation is associated not only with the diversity of animal species with different immune systems but also with delays in the development of economical, and robust formulations (low toxicity, generate a potent immune response, optimum antigen adsorption-dsorption capacity, biodegradable, and stable under normal storage conditions) that comply with good production practices (48, 49). Despite this, the industry remains very attentive to the evaluation of these substances because their incorporation has many advantages: optimization of the immunogenic efficacy of current vaccines, increase the immune response at mucosal level without the need for injection, production of larger quantities of doses with fewer antigens, and reduction of production costs without sacrificing the quality of the vaccine (47).

Another coincidental feature identified in this study is the dominance of avian, and swine vaccines both in scientific articles and in the market. This occurrence, although observed in a previous study on veterinary infectious diseases (23), and confirms once again that science and industry continue to focus on the immunoprotection of fundamental species in the production of animal protein for the human consumption, promoting the livelihood of rural poor, especially in underdeveloped countries, and supporting national food security (30).

Together with the identification of common interests between science and industry, divergent thematic areas were also found. In the first case, there was no correspondence between the high number of co-occurrences identified in the bibliometric study (*n* = 720) and the scarce presence of live vaccines in the market (0.7%, 1/136, Flu Avert®, H3N8). This fact suggests that science insists on achieving modified live strains (less virulent or avirulent) despite serious safety issues in clinical use and cumbersome manufacturing processes. This research may be encouraged by the fact that these are the only vaccines that best mimic natural infection, thus inducing strong humoral and cellular immunity. Several attenuation options have been explored, such as degradation of viral proteins (50), deletion of the NS1 gene encoding non-structural protein 1, or point mutations in the haemagglutinin gene (51–55). Although the results are successful in generating robust humoral, mucosal and cellular immunity against homologous and heterologous viruses, the resulting genetic constructs have not progressed beyond the scientific frontier. These options have yet to overcome numerous regulatory barriers and to implement new vaccination strategies involving the simultaneous use of live and inactivated vaccines.

In the second case, the numerous co-ocurrent publications on canine vaccination against avian influenza (*n* = 510) and the poor presence of commercial vaccines may be related to the gradual changes taking place in our societies regarding pet ownership and care. The loss of traditional pet functions such as protection, hunting and defense to contemporary functions such as companionship and provision of affection (56, 57) encourages pet adoption, cohabitation in smaller spaces and sharing of pathogens. The increased likelihood of zoonotic events, coupled with frequent reports of influenza infections of various subtypes in dogs (58), raise new concerns that necessitate further research into immunoprotection. However, this scientific interest has not yet translated into vaccine production, and dogs are the least represented species in the global market, with only 4.5% of commercial vaccines available in 2023.

The final area of divergence identified in this study concerns equine vaccines. In this respect, little scientific interest in research into new equine flu vaccines was observed (*n* = 19), despite the fact that equine flu is a species of great military, cultural, genetic, sporting, recreational and economic value. This reality is contradicted by the large number of commercial vaccines available on the world market (34.5%, 47/136), being the second most immunoprotected animal species. This imbalance between science and industry is difficult to explain considering the great usefulness of the horse for humans since ancient times (transportation, agricultural and livestock work, meat, milk, hair, and leather) and the great impact of the disease on the equine industry (compulsory health declaration, low sporting performance and restrictions on international trade) (59, 60).

With respect to the significant genetic diversity of influenza strains (high and low pathogenicity subtypes) involved in research and commercial vaccine production was an expected occurrence, given the extensive antigenic variants observed in wild strains (61). Consequently, it is not coincidental to find close links between the field strains with the highest global circulation and vaccine strains (62), in particular those with a high capacity to rapidly spread between migratory birds, domestic birds, mammals and humans such as H3Nx, and H5Nx. The industrial use of numerous lineages responsible for outbreaks in Africa, Asia, America and Europe is extremely important to increase the effectiveness of disease prevention and control programs in countries that practice vaccination and reduce the frequency of animals with severe forms of the disease (63–65).

The unstable health situation associated with the constant genetic evolution of the pathogen, the increasing number of affected species and the ‘One Health’ approach will encourage the veterinary and human scientific and industrial communities to continue their collaboration. Unity of public and private interests will be imperative not only for effective, coordinated and sustained epidemiological surveillance and reporting actions worldwide, but also for rapid dissemination of knowledge on new influenza A virus sequences and outbreaks, in order to prevent possible future pandemics (66, 67).

The positive impact observed in those countries that decided to implement massive vaccination (66), may change in the future. The rapid antigenic evolution of the virus, chemical instability of hemagglutinin/neuraminidase, and wide range of susceptible and transmitting hosts make it almost impossible to predict the subtype of the next pandemic virus and, therefore, the existence of an adequate vaccine (12, 67). Faced with these questions, science is attempting to design so-called “universal vaccines” that include several subtypes of influenza in the same formulation. In this regard, in vivo tests using H1N9/H3N8/H5N1/H7N3 mixtures yield satisfactory results; however, there is still a long way to go (68). The efficacy and safety of these vaccines depend on many factors: antigenic match of vaccine strains to circulating strains, manufacturing procedures, adjuvants, antigen concentration, dosage and method of administration (69). Therefore, these formulations cannot be expected to be applied in daily clinical practice in the short term, which justifies their peripheral position observed in Figure 1. The success of this new immunoprotective option is closely linked to the union of public and private interests. The resulting alliance will allow not only effective, coordinated and sustained epidemiological surveillance, but also rapid dissemination of emerging epidemic strain sequences for inclusion in new formulations (70, 71).

The extensive network of academic journals publishing veterinary scientific results on avian influenza, the significant participation of countries in joint research (Figure 2) and the large number of commercial vaccines worldwide (Table 2) reveal a reality: scientists, vaccine manufacturers and farmers are jointly searching for answers to defeat the same adversary. All, from different approaches, identify this virus as a major threat to the security of nations and urgently need to reduce the serious health and economic impacts that the disease can cause (increased mortality, reduced production and trade restrictions) (72). The high international priority given to this virus (7, 8) may have been an element promoting the balance observed between academic and industrial production of veterinary vaccines in some of the countries involved in this study, such as the United States, Vietnam, Iran, Spain, Germany, Russia, the Netherlands, and Egypt.

This heterogeneity is due to the wide geographical distribution of the disease, its great epidemiological complexity, the increasing number of affected species (1, 2), the experience in research, development and application of vaccines that some countries possess (73), as well as the existence of an economic cooperation framework that prioritizes cross-border research between developing nations and high-income international scientific institutions (74). In this context, the prominent presence of the United States and China coincides with the largest epidemic events reported globally and therefore, they are the most interested in collaborating with other nations to mitigate zoonotic risks and the resulting economic and trade impacts (62, 64). For these reasons, they dedicate significant research funds resulting in a high volume of publications and international collaborations, as illustrated in Figure 3 (75).

The commercial interest of pharmaceutical companies in introducing influenza vaccines combined with antigens from different subtypes (18.4%, 25/136), viruses, and bacterias (52.2%, 71/136) to the market arises from multiple reasons. These include extending protection against several subtypes in a single administration (76), preventing a greater number of diseases per dose, rapid compliance with the vaccination schedule, and increased immunization coverage. Other elements favoring the presence of these formulations on the world market are the constant emergence of antibiotic-resistant bacterial strains (77), co-circulation of different pathogens in the same population, reduced costs in application, transportation, and storage of biological material as well as decreased stress in animals due to less manipulation (78). Therefore, these formulations are highly appreciated by animal health professionals and prove highly useful in the case of avian influenza. Recent evidence confirms that this pathogen has the capacity to co-infect animals, increasing the frequency and severity of respiratory tract lesions (79). Based on this evidence, combination vaccines emerge as an appropriate solution, eliciting a rapid and improved immune response in both avian and equine species compared to monovalent vaccines against Newcastle and herpesvirus types 1 and 4 (24, 80, 81).

## 5 Limitations and strengths of the study

This study has several limitations in its implementation. First, Scopus was the sole database consulted, and only articles written in English were considered. This could potentially lead to an underestimation of the available information, as other important databases and languages used to communicate scientific findings were not included. However, these decisions were made considering that Scopus constitutes the largest database of peer-reviewed scientific literature globally (22) and that the main international journals (Q1 and Q2) exclusively accept articles in English (70, 71, 82). Additionally, articles written in other languages (Spanish, Portuguese, French, German, Russian, and Chinese, among others) tend to receive fewer citations, despite being indexed in international databases (83). Second, the bibliometric search may have overestimated the productivity of certain countries because the primary authors’ affiliation does not always reflect their true institution of origin or the place where the research was conducted. Third, by analyzing 24 companies from 17 countries, it was possible to exclude others of regional importance, thus potentially causing biases or data omissions.

However, the results shown include formulations from the largest commercial players in the global veterinary biopharmaceutical sector, such as Boehringer Ingelheim, Elanco, MSD Animal Health, and Zoetis. Despite all these limitations, the study can be used to identify global trends in veterinary biopharmaceutical science and industry for preventing avian influenza. Furthermore, it can serve as a starting point for future research on this topic, involving similar or different productive and affective species, thereby addressing the identified limitations.

## 6 Conclusion

The study demonstrated the fundamental role of classical production methods (based on the use of the whole pathogen) in avian influenza A research and the production of veterinary vaccines.

## Data Availability

The original contributions presented in the study are included in the article/supplementary material, further inquiries can be directed to the corresponding author.

## Footnotes

^1^ https://www.scimagojr.com
